# The Rural-Urban Difference in BMI and Anemia among Children and Adolescents

**DOI:** 10.3390/ijerph13101020

**Published:** 2016-10-18

**Authors:** Yan Zou, Rong-Hua Zhang, Shi-Chang Xia, Li-Chun Huang, Yue-Qiang Fang, Jia Meng, Jiang Chen, He-Xiang Zhang, Biao Zhou, Gang-Qiang Ding

**Affiliations:** Zhejiang Provincial Center for Disease Control and Prevention, 3399 Binsheng Road, Hangzhou 310051, China; zouyan0573@163.com (Y.Z.); yingyang001@yeah.net (R.-H.Z.); yingyang905@yeah.net (L.-C.H.); yingyang907@yeah.net (Y.-Q.F.); difang9013@yeah.net (J.M.); yingyang906@yeah.net (J.C.); yingyang921@yeah.net (H.-X.Z.); yingyang910@yeah.net (B.Z.); yingyang901@yeah.net (G.-Q.D.)

**Keywords:** obesity, wasting, anemia, children, adolescent

## Abstract

There is growing concern over the double burden of over- and under-nutrition in individuals, especially in children and adolescents, which could dwarf their growth and development. This study aims to explore the rural-urban difference in BMI and anemia among children and adolescents. A stratified cluster sampling technique was employed. Dietary data were collected through interviews, and anthropometric values were measured. There were 1534 children and adolescents who participated in this study, including 775 male and 759 female participants. The prevalence of obesity among children living in a city, township and rural area was 10.3%, 8.5% and 5.5%, and that among adolescents was 1.4%, 2.9% and 2.8%. The prevalence of anemia among children and living in a city, township and rural area was 4.3%, 2.5% and 4.5%, while that among adolescents was 6.1%, 3.7% and 11.3%, respectively, with significant difference (χ^2^ = 10.824, *p* = 0.004). The prevalence of being overweight, obesity and anemia was significant when comparing children with adolescents (χ^2^ = 37.861, *p* = 0.000; χ^2^ = 19.832, *p* = 0.000; χ^2^ = 8.611, *p* = 0.003). Findings of this study indicate the double burden of malnutrition in Zhejiang province, characterized by a high prevalence of being overweight, obesity and anemia among children and a high prevalence of anemia among adolescents living in townships.

## 1. Introduction

Obesity in adolescents and children has become a global public health problem. The worldwide prevalence of being overweight and obesity for children and adolescents has increased dramatically in these past decades, which led to associated health risks increasing and considerable health care costs increasing [1,2]. In 2004, according to IOTF criteria, it was estimated that 10% of children worldwide aged 5–17 years were overweight and that 2%–3% were obese [3]. In China, Zhang reported that the prevalence of combined overweight and obesity increased from 1.79% and 1.66% in 1985 to 31.12% and 20.11% in 2014 for boys and girls, respectively [4].

Despite the nutrition transition and improved nutritional status, wasting and anemia remain a major public health problem. Children are also the vulnerable group for iron and zinc deficiencies, and their nutritional status is a sensitive indicator of community health and nutrition. In China, more than 15% of the population was anemic in 2002 [5], while iron deficiency is the main cause of anemia in developing countries [6]. As the traditional Chinese diet is low in meat and milk and high in vegetable and other plant foods, the iron bioavailability is low [7].

Whether under-nutrition or over-nutrition is associated with increased risk of anemia remains controversial. For example, obesity may cause anemia in several ways, for example by low-grade inflammation and relative iron deficit [8]; while other literature reported that women with obesity were less likely to be anemic [9]. In addition, Conde reported that no association between anemia and any other child anthropometric indicator was detected [10], and Laillou demonstrated that micronutrient deficiencies are an issue across the weight spectrum among women in Vietnam, with only vitamin A status being better among overweight than underweight women [11].

There is growing concern over the double burden of over- and under-nutrition in individuals, especially in children and adolescents, which could dwarf their growth and development and result in susceptibility to chronic disease and reduced well-being. This paper aims to explore the rural-urban difference in BMI and anemia among children and adolescents and to compare the daily nutrient intake among children and adolescents with overweight, obesity or wasting, as well as to explore if it could be likely related with low or high daily nutrient intake among children and adolescents with or without malnutrition.

## 2. Materials and Methods

### 2.1. Participants

**Figure 1:**
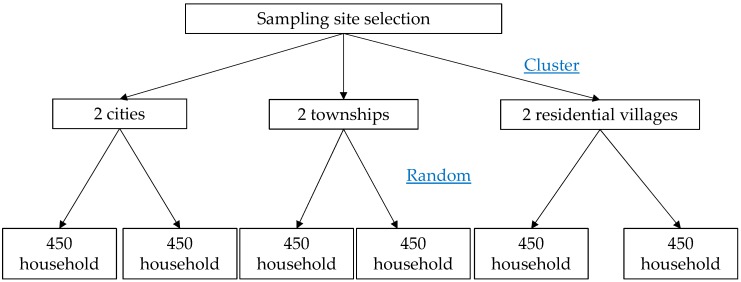
Flow chart of the sampling process.

This survey is part of the Chinese national nutrition survey during 2010–2012. A stratified cluster sampling technique was employed in the present cross-sectional study. Based on socioeconomic characteristics, 2 cities, 2 townships and 2 residential villages were randomly selected as where the investigation was conducted. In every sampling unit, 450 households were selected by random a sampling method according to the household registration information. Then, every member of the sampled household was interviewed. Figure 1 presents the flow chart of the sampling process. Children (7–12 years old) and adolescents (13–17 years old) were the subjects of this study. The response rate for households was 95%, and the response rate of each family member in the response household was 100%.

A questionnaire was used to obtain general personal information, which covered the information of gender, age, residence and information of dietary intake. Energy and nutrient intake was calculated using three consecutive days of 24-h dietary recall in conjunction with the China Food Composition Table published in 2002 [12]. The questionnaire was administrated face to face by trained staff through door to door interviews. The dietary recall was collected from the parents on behalf of their children, while it was collected from adolescents themselves. The three consecutive days only cover the weekdays (from Wednesday–Friday).

### 2.2. Measurements and Definition

Height was measured without shoes to the nearest 0.2 cm using a portable SECA stadiometer, and weight was measured without shoes and overcoat to the nearest 0.1 kg on a calibrated beam scale. BMI was calculated by weight (kg)/height (m)^2^. Overweight and obesity were defined by the BMI cut-off points recommended in 2002 by Group of China Obesity Task Force (Group of China Obesity Task Force 2004). Additionally, wasting of children and adolescents was defined by the BMI cut-off points according to the screening standard for malnutrition (WS/T 456-2014).

Plasma Hb was determined by the cyanmethemoglobin determination method. Anemia was defined by the Hb cut-off points recommended in 2001 by WHO and UNICEF. The cut-off points (g/L) were 115, 120, 120 and 130 (g/L) for children aged 5–11, 12–14, older than 15 years (girls) and older than 15 years (boys).

### 2.3. Ethics

Research protocols were approved by Zhejiang Provincial Center for Disease Control and Prevention (Ethic approval code: T-043-R-2010). All subjects or their guardian provided written informed consent after the research protocols were carefully explained to them. Thus, informed consents from the parents/guardians of all participants under the age of 16 were also received.

### 2.4. Statistics 

As continuous variables were not normally distributed, they were described as the median, 25th and 75th percentiles. The differences of quantitative data between the two groups were evaluated by the chi square test. The differences of dietary nutrient intake between children and adolescents with or without malnutrition were evaluated by the nonparametric test (Kruskal–Wallis test). Data processing and statistical analyses were performed using SAS9.2 software (SAS Institute, Cary, NC, USA). All tests were two-sided, and the level of significance was set at *p* < 0.05.

## 3. Results

There were 1534 children and adolescents who participated in this study, including 775 male and 759 female participants. The percent of participants from a city, township and rural area was 26.7%, 37.0% and 36.4%, respectively.

The prevalence of wasting among children living in a city, township and rural area was 5.2%, 8.6% and 9.7%, respectively, with no significant difference (χ^2^ = 3.749, *p* = 0.153), and that among adolescents was 9.5%, 9.1%, 10.9%, respectively, with no significant difference (χ^2^ = 0.472, *p* = 0.790). The prevalence of wasting among male children was 9.2%, higher than that of females (6.6%), with no significant difference (χ^2^ = 1.870, *p* = 0.171), while that among male adolescents was 13.0%, higher than that of female (6.6%), with a significant difference (χ^2^ = 7.293, *p* = 0.007). The prevalence of obesity among children living in a city, township and rural area was 10.3%, 8.5% and 5.5%, respectively, with no significant difference (χ^2^ = 4.544, *p* = 0.103), and that among adolescents was 1.4%, 2.9% and 2.8%, respectively, also with no significant difference (χ^2^ = 1.037, *p* = 0.595). The prevalence of obesity among male children was 10.7%, higher than that of females (5.2%), with a significant difference (χ^2^ = 8.519, *p* = 0.004), but there was no significance among male adolescents (3.3%) and female adolescents (1.6%) (χ^2^ = 1.819, *p* = 0.177). The prevalence of anemia among adolescents living in a city, township and rural area was 6.1%, 3.7% and 11.3%, respectively, with a significant difference (χ^2^ = 10.824, *p* = 0.004), but there was no significant difference among children living in a city, township and rural area (χ^2^ = 1.955, *p* = 0.376). The prevalence of anemia among male adolescents was 4.2%, lower than that of females (10.5%), with a significant difference (χ^2^ = 9.342, *p* = 0.002), but the prevalence of anemia among male children (4.0%) and female children (3.6%) was not significant (χ^2^ = 0.103, *p* = 0.748) (Table 1).

**Figure 2:**
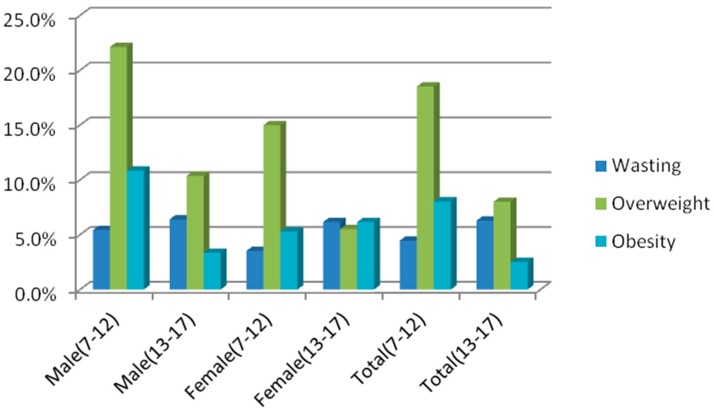
The percentages of population from wasting to obese.

Figure 2 shows the percentages of population from wasting to obese. The prevalence of overweight, obesity and anemia was significant when comparing children with adolescents (χ^2^ = 37.861, *p* = 0.000; χ^2^ = 19.832, *p* = 0.000; χ^2^ = 8.611, *p* = 0.003), while that of wasting was not significant (χ^2^ = 1.801, *p* = 0.180) (Table 2 and Table 3).

The prevalence of anemia among children and adolescents with being overweight, obesity, wasting and a reasonable BMI (the BMI is in the range of critical value for wasting and being overweight) was 1.46%, 4.55%, 7.50% and 5.53%. There was no significant difference on the prevalence of anemia between children and adolescents with different BMI conditions (χ^2^ = 5.084, *p* = 0.166) (Table 4).

### Daily Nutrient Intake among Children and Adolescents with Wasting, Reasonable BMI, Overweight and Obesity in Zhejiang Province

The median of the daily intake of protein among children and adolescent with wasting, reasonable BMI, overweight and obesity was 39.89 g, 48.31 g, 46.49 g, 29.09 g, respectively, with a significant difference (Z = 8.015, *p* = 0.046) (Table 5). The median of daily intake of fat from wasting to obese was 50.35 g, 52.59 g, 51 g, 38.52 g, respectively, with a significant difference (Z = 10.211, *p* = 0.017). In addition, the median of daily intake of riboflavin and phosphorus among children and adolescents from wasting to obese was 0.4 mg and 518.84 mg, 0.51 mg and 690.35 mg, 0.5 mg and 620.37 mg, 0.51 mg and 425.55 mg, respectively, with a significant difference (Z = 8.366, *p* = 0.039; Z = 7.886, *p* = 0.048).

## 4. Discussion

On the basis of this cross-sectional survey, this paper reports on the prevalence of wasting, overweight, obesity and anemia among children and adolescents living in a city, township and rural area and provides evidence linking specific dietary factors related to malnutrition in this population group. Since successful malnutrition prevention strategies should rely on evidence-based public health approaches, the results of this paper could represent suggestions for effective interventions and policies aiming at a target age group and region for curbing the epidemic of malnutrition in youth.

A study carried out in Zhejiang province in 2002 suggested that in urban areas of eastern China, a dual picture is emerging with the problems of excess (overweight and obesity) coexisting with underweight and anemia [13]. This study was carried out during 2010–2012, and suggested that obesity also coexists with wasting and anemia; however, the prevalence rates of obesity, overweight, wasting and anemia were lower than another study carried out in Huai’an and Nanjing [14], but the difference is there were significant differences of anemia prevalence between urban and rural areas other than the previously reported non-significant urban-rural difference. The potential causes of urban-rural differences may be related to the socioeconomic conditions. Children and adolescents living in urban areas often live with better socioeconomic conditions and less physical activity, as well as different dietary patterns, compared with those living in rural areas, which was consistent with our previous reported study among adults [15]. The findings of this study indicate the double burden of malnutrition in Zhejiang province, characterized by a high prevalence of overweight, obesity and anemia among children and a high prevalence of anemia among adolescents living in townships. Olaya reported that younger child and boys were more affected no matter which standard we used [16]. This decline may be due partly to the attenuation of the positive energy, balanced during puberty or a possible positive trend of increased weight across generations due to exposure to environmental factors earlier in life. In agreement with previous studies [17], we observed a greater prevalence of obesity in male children and adolescents than in female children and adolescents. Gender-specific differences may be explained by the greater lean mass associated with a muscular male body build and bone mass. Excess weight-for-height attributable to lean and bone tissue rather than body fat may, in part, account for the observed high prevalence of overweight men according to BMI criteria [18].

Obesity is the consequence of a long-term imbalance between energy intake and energy expenditure. However, with cross-sectional studies, it is not possible to measure such long-term discrepancies in energy balance. In this study, the distribution of the daily intake of proteins and fat was different among children and adolescent with wasting, reasonable BMI, overweight and obesity. The differentials between children and adolescents with or without malnutrition may be explained by differences in dietary patterns and food choices; this is consistent with previous studies that increased inappropriate eating behaviors significantly have effects on body composition [19,20]. In this study, the prevalence of anemia between children and adolescents being overweight, obesity, wasting and a reasonable BMI were similar with no significant difference. The causes of anemia are multi-factorial, and the nutritional factors include inadequate intake of iron-dense foods, consumption of staple foods that are poor iron sources or that contain iron absorption inhibitors [21] and vitamin deficiencies [22]. Sharif reported that ferritin concentrations were similar in both obese and non-obese Children [23]. Additionally, Cepeda-Lopez reported that sharply higher rates of iron deficiency in obese children are predicted by obesity-related inflammation rather than by differences in dietary iron intake [24]. Thus, children and adolescents with obesity were less likely or more likely to be anemic as compared to those without obesity. Key behavioral risk factors in the increase of obesity also include a low consumption of fruits and vegetables; while in this study, there were no significant difference on nutrient intake between children and adolescents, except protein, fat, phosphorus and riboflavin.

This study has several limitations. The present study is a cross-sectional design that disallows a sequence of temporality to be established for malnutrition and dietary patterns. Data on dietary intake were collected by three consecutive days with the 24-h dietary recall method, and recall bias may exist.

## 5. Conclusions

In conclusion, the prevalence of obesity among children living in a city, township and rural area was 10.3%, 8.5% and 5.5%, and that among adolescents was 1.4%, 2.9% and 2.8%. The prevalence of wasting among children living in a city, township and rural area was 5.2%, 8.6% and 9.7%, and that among adolescents was 9.5%, 9.1%, 10.9%. The prevalence of anemia among children and living in a city, township and rural area was 4.3%, 2.5% and 4.5%, while that among adolescents was 6.1%, 3.7% and 11.3%. Findings of this study indicate the double burden of malnutrition in Zhejiang province, characterized by a high prevalence of overweight, obesity and anemia among children and a high prevalence of anemia among adolescents living in townships.

## Figures and Tables

**Table 1:** The percentages of wasting, obesity and anemia among children and adolescents living in city, township and residential village.

Nutritional Status	Population	City	Township	Residential Village	χ^2^	*p*
Wasting	Children	5.2%	8.6%	9.7%	3.749	0.153
	Adolescents	9.5%	9.1%	10.9%	0.472	0.790
Obesity	Children	10.3%	8.5%	5.5%	4.544	0.103
	Adolescents	1.4%	2.9%	2.8%	1.037	0.595
Anemia	Children	4.3%	2.5%	4.5%	1.955	0.376
	Adolescents	6.1%	3.7%	11.3%	10.824	0.024

**Table 2:** The distribution of wasting, overweight and obesity in Zhejiang province stratified by age and gender.

Age (Years)	Male							Female							Total						
	Wasting		Overweight		Obesity			Wasting		Overweight		Obesity			Wasting		Overweight		Obesity		
	N	Percentage	N	Percentage	N	Percentage	Total	N	Percentage	N	Percentage	N	Percentage	Total	N	Percentage	N	Percentage	N	Percentage	Total
7	4	4.82%	17	20.48%	9	10.84%	83	4	4.12%	16	16.49%	7	7.22%	97	8	4.44%	33	18.33%	16	8.89%	180
8	4	5.13%	26	33.33%	16	20.51%	78	2	2.78%	17	23.61%	6	8.33%	72	6	4.00%	43	28.67%	22	14.67%	150
9	6	9.84%	14	22.95%	8	13.11%	61	2	2.90%	12	17.39%	5	7.25%	69	8	6.15%	26	20.00%	13	10.00%	130
10	3	3.95%	16	21.05%	7	9.21%	76	2	2.63%	8	10.53%	1	1.32%	76	5	3.29%	24	15.79%	8	5.26%	152
11	5	6.94%	13	18.06%	2	2.78%	72	2	2.74%	7	9.59%	3	4.11%	73	7	4.83%	20	13.79%	5	3.45%	145
12	2	2.74%	12	16.44%	6	8.22%	73	4	5.97%	8	11.94%	2	2.99%	67	6	4.29%	20	14.29%	8	5.71%	140
13	4	5.56%	6	8.33%	2	2.78%	72	7	12.28%	3	5.26%	1	1.75%	57	11	8.53%	9	6.98%	3	2.33%	129
14	7	10.77%	5	7.69%	1	1.54%	65	3	4.84%	0	0.00%	0	0.00%	62	10	7.87%	5	3.94%	1	0.79%	127
15	3	4.62%	8	12.31%	2	3.08%	65	4	5.56%	7	9.72%	2	2.78%	72	7	5.11%	15	10.95%	4	2.92%	137
16	4	5.97%	6	8.96%	3	4.48%	67	2	2.86%	3	4.29%	1	1.43%	70	6	4.38%	9	6.57%	4	2.92%	137
17	3	5.08%	9	15.25%	3	5.08%	59	3	6.25%	4	8.33%	1	2.08%	48	6	5.61%	13	12.15%	4	3.74%	107
Total (7–12)	24	5.42%	98	22.12%	48	10.84%	443	16	3.52%	68	14.98%	24	5.29%	454	40	4.46%	166	18.51%	72	8.03%	897
Total (13–17)	21	6.40%	34	10.36%	11	3.35%	328	19	6.15%	17	5.50%	5	6.15%	309	40	6.28%	51	8.00%	16	2.51%	637

**Table 3:** The distribution of hemoglobin and anemia in Zhejiang province stratified by age and gender.

Age (Years)	Male				Female				Total				
	Hemoglobin (g/L)	SD	Anemia (N, %)		Hemoglobin (g/L)	SD	Anemia (N, %)		Hemoglobin (g/L)	SD	Anemia (N, %)		Total
7	132.33	10.06	4	4.80%	133.33	13.16	4	4.10%	132.86	11.78	8	4.40%	180
8	135.92	13.00	4	5.10%	134	13.02	5	6.90%	134.95	13.00	9	6.00%	150
9	135.97	10.33	2	3.30%	135.62	11.15	1	1.40%	135.78	10.74	3	2.30%	130
10	138.56	9.54	0	0.00%	138.25	10.07	1	1.30%	138.32	9.79	1	0.70%	152
11	136.55	11.04	2	2.80%	139.6	10.11	0	0.00%	138.06	10.66	2	1.40%	145
12	139.81	12.14	6	8.20%	140.4	11.54	5	7.50%	138.81	11.83	11	7.90%	140
13	143.84	14.47	4	5.60%	137.16	10.28	3	5.30%	140.91	13.18	7	5.40%	129
14	143.25	16.22	5	7.70%	135.43	10.96	4	6.50%	139.61	14.49	9	7.10%	127
15	154.56	18.45	4	6.20%	137.89	28.18	10	13.90%	145.59	25.49	14	10.22%	137
16	153.71	10.33	0	0.00%	135.58	14.77	10	15.20%	144.06	15.72	10	7.29%	137
17	151.64	14.00	1	1.70%	133.37	13.85	5	10.40%	143.35	16.60	6	5.60%	107
Total (7–12)	135.78	11.17	18	3.98%	136.19	11.98	16	3.55%	136.00	11.59	34	3.79%	897
Total (13–17)	149.18	15.59	14	4.21%	136.07	17.61	32	10.49%	142.77	17.84	46	7.22%	637

**Table 4:** The prevalence of anemia stratified by gender and BMI in Zhejiang province.

	Anemia	Overweight		Obesity		Overweight		Obesity		χ^2^	*p*
		N	Percentage	N	Percentage	N	Percentage	N	Percentage		
Male	Yes	2	4.44%	28	4.74%	0	0.00%	2	3.39%	4.091	0.252
	No	43	95.56%	563	95.26%	80	100.00%	57	96.61%		
Female	Yes	2	6.90%	4	11.43%	40	6.27%	2	3.51%	2.321	0.508
	No	27	93.10%	31	88.57%	598	93.73%	55	96.49%		
Total	Yes	6	7.50%	68	5.53%	2	1.46%	4	4.55%	5.084	0.166
	No	74	92.50%	1161	94.47%	135	98.54%	84	95.45%		

note: Chi-square tests.

**Table 5:** Daily nutrient intake among children and adolescents with wasting, reasonable BMI, overweight and obesity in Zhejiang province.

Nutrients/Day	Wasting (*N* = 80)			Reasonable BMI (*N* = 1149)			Overweight (*N* = 217)			Obesity (*N* = 88)				
	Median	25%	75%	Median	25%	75%	Median	25%	75%	Median	25%	75%	H	*p*
Energy (kcal)	1307.83	1059.37	1408.04	1415.83	1144.24	1758.06	1415.3	1272.91	1758.06	848.86	396.47	1301.24	6.534	0.088
Protein (g)	39.89	28.21	46.21	48.31	35.47	62.31	46.49	40.4	49.29	29.09	11.03	47.15	8.015	0.046
Fat (g)	50.35	22.95	58.04	52.59	33.26	80.87	51	43.29	67.93	38.52	11.1	65.93	10.211	0.017
Carbohydrate (g)	160.89	134.47	198.62	182.42	149.09	234.11	179.07	155.69	234.53	93.43	53.79	133.06	1.241	0.743
Cholesterol (mg)	99.96	44.22	365.79	140.55	89.51	330.56	164.47	121.92	300.86	169.6	72.84	266.36	4.025	0.259
Dietary fiber (g)	4.76	4.4	6.28	5.46	3.91	9.97	10.05	8.02	11.74	2.48	1.54	3.42	6.574	0.087
Vitamin A (μg RE)	174.17	103.12	294.43	226.8	108.45	326.96	231.44	143.05	308.16	512.94	60.36	965.53	1.744	0.627
Retinol (μg RE)	34.89	20.04	139.91	50.25	23.75	108.6	59.36	33.14	181.36	457.56	25.11	890.01	5.907	0.116
Thiamin (mg)	0.46	0.34	0.53	0.58	0.43	0.73	0.53	0.45	0.55	0.37	0.16	0.58	5.859	0.119
Riboflavin (mg)	0.4	0.32	0.53	0.51	0.32	0.66	0.5	0.44	0.55	0.51	0.12	0.9	8.366	0.039
Niacin (mg NE)	9.39	7.43	11.67	9.53	7.91	13.51	9.08	8.23	10.67	9.11	3.15	15.07	3.861	0.277
Vitamin C (mg)	28.33	21.65	49.11	27.88	17.43	46.45	29.1	22.61	31.06	14.24	9.7	18.78	4.446	0.217
Vitamin E (mg)	8.55	7.85	19.38	15.37	9.42	23.56	16.39	12.44	18.19	6.31	2.5	10.11	6.435	0.092
Ca (mg)	218.89	156.18	288.5	240.45	148.53	346.04	214.69	170.31	242.63	101.69	58.63	144.75	3.559	0.313
P (mg)	518.84	388.44	639.16	690.35	466.93	874.84	620.37	572.42	626.39	425.55	154.27	696.83	7.886	0.048
K (mg)	814.06	642.08	1145.42	1135.84	678.08	1622.07	1221.53	1052.81	1363.47	619.26	289.41	949.11	3.58	0.311
Na (mg)	2623.96	1236.4	4179.63	3360.17	1972.54	4599.96	2990.45	2398	4960.4	2259.08	731.73	3786.43	1.84	0.606
Mg (mg)	156.16	125.04	175.45	179.94	134.16	240.09	163.08	143.08	208.88	100.48	47.51	153.45	2.941	0.401
Fe (mg)	12.95	11.49	14.9	13.72	10.81	17.3	13.08	11.07	14.68	12.13	3.3	20.97	2.297	0.513
Zn (mg)	6.73	5.08	7.6	7.89	6.18	9	7.24	6.1	7.75	4.62	1.68	7.55	3.97	0.265
Se (μg)	18.04	13.55	28.42	21.98	17.77	41.78	23.99	19.56	30.31	29.61	9.56	49.66	7.645	0.054
Cu (mg)	1.04	1	1.35	1.32	0.98	1.77	1.55	1.31	3.04	0.6	0.28	0.91	4.545	0.208
Mn (mg)	3.3	2.46	3.68	3.89	3.39	4.53	3.54	2.73	4.35	2.48	0.88	4.07	3.662	0.3

## References

[1] Weiss R., Dziura J., Burgert T.S., Tamborlane W.V., Taksali S.E., Yeckel C.W., Allen K., Lopes M., Savoye M., Morrison J. (2004). Obesity and the metabolic syndrome in children and adolescents. N. Engl. J. Med..

[2] Fu J.F., Liang L., Zou C.C., Hong F., Wang C.L., Wang X.M., Zhao Z.Y. (2007). Prevalence of the metabolic syndrome in Zhejiang Chinese obese children and adolescents and the effect of metformin combined with lifestyle intervention. Int. J. Obes..

[3] Lobstein T.B.L., Uauy R. (2004). Obesity in children and young people: A crisis in public health. Obes. Rev..

[4] Zhang Y.X. (2016). Changes in the nutritional status of children and adolescents in Shandong, China. Public Health Nutr..

[5] Li L.M., Rao K.Q., Kong L.Z., Yao C.H., Xiang H.D., Zhai F.Y., Ma G.S., Yang X.G. (2005). Technical working group of China national nutrition and health survey: A description on the Chinese national nutrition and health survey in 2002. Chin. J. Epidemiol..

[6] Zimmermann M.B., Hurrell R.F. (2007). Nutritional iron deficiency. Lancet.

[7] Hurrell R., Egli I. (2010). Iron bioavailability and dietary reference values. Am. J. Clin. Nutr..

[8] Winther S.A., Finer N., Sharma A.M., Torp-Pedersen C., Andersson C. (2014). Association of anemia with the risk of cardiovascular adverse events in overweight/obese patients. Int. J. Obes..

[9] Qin Y., Melse-Boonstra A., Pan X., Yuan B., Dai Y., Zhao J., Zimmermann M.B., Kok F.J., Zhou M., Shi Z. (2013). Anemia in relation to body mass index and waist circumference among Chinese women. Nutr. J..

[10] Conde W.L., Monteiro C.A. (2014). Nutrition transition and double burden of undernutrition and excess of weight in Brazil. Am. J. Clin. Nutr..

[11] Laillou A., Yakes E., Le T.H., Wieringa F.T., Le B.M., Moench-Pfanner R., Berger J. (2014). Intra-individual double burden of overweight and micronutrient deficiencies among Vietnamese women. PLoS ONE.

[12] Yang Y., Wang G., Pan X. (2002). China Food Composition Table 2002.

[13] Gao G., Zuo P., Sun G., Kai H. (2009). Study on nutritional status of primary and middle school students in Huai’an and Nanjing. J. Hyg. Res..

[14] Hesketh T., Ding Q.J., Tomkins A.M. (2002). Disparities in economic development in Eastern China: Impact on nutritional status of adolescents. Public Health Nutr..

[15] Zou Y., Zhang R., Zhou B., Huang L., Chen J., Gu F., Zhang H., Fang Y., Ding G. (2015). A comparison study on the prevalence of obesity and its associated factors among city, township and ruralarea adults in China. Br. Med. J..

[16] Olaya B., Moneta M.V., Pez O., Bitfoi A., Carta M.G., Eke C., Goelitz D., Keyes K.M., Kuijpers R., Lesinskiene S. (2015). Country-level and individual correlates of overweight and obesity among primary school children: A cross-sectional study in seven European countries. BMC Public Health.

[17] Barbu C.G., Teleman M.D., Albu A.I., Sirbu A.E., Martin S.C., Bancescu A., Fica S.V. (2015). Obesity and eating behaviors in school children and adolescents- data from a cross sectional study from Bucharest, Romania. Biol. Med. Cent. Public Health.

[18] Steinberger J., Jacobs D.R., Raatz S., Moran A., Hong C.P., Sinaiko A.R. (2005). Comparison of body fatness measurements by BMI and skinfolds vs dual energy X-ray absorptiometry and their relation to cardiovascular risk factors in adolescents. Int. J. Obes..

[19] Nasreddine L., Naja F., Akl C., Chamieh M.C., Karam S., Sibai A.M., Hwalla N. (2014). Dietary, lifestyle and socio-economic correlates of overweight, obesity and central adiposity in Lebanese children and adolescents. Nutrients.

[20] Patrick K., Norman G.J., Calfas K.J., Sallis J.F., Zabinski M.F., Rupp J., Cella J. (2004). Diet, physical activity, and sedentary behaviors as risk factors for overweight in adolescence. Arch. Pediatr. Adolesc. Med..

[21] World Health Organization (2001). Iron Deficiency Anemia: Assessment, Prevention, and Control.

[22] Fishman S.M., Christian P., West K.P. (2000). The role of vitamins in the prevention and control of anemia. Public Health Nutr..

[23] Sharif M., Madani M., Tabatabaie F. (2014). Comparative evaluation of iron deficiency among obese and non-obese children. Iran. J. Ped. Hematol. Oncol..

[24] Cepeda-Lopez A.C., Osendarp S.J., Melse-Boonstra A., Aeberli I., Gonzalez-Salazar F., Feskens E., Villalpando S., Zimmermann M.B. (2011). Sharply higher rates of iron deficiency in obese Mexican women and children are predicted by obesity-related inflammation rather than by differences in dietary iron intake. Am. J. Clin. Nutr..

